# Near-Complete Human Sapovirus Genome Sequences from Kenya

**DOI:** 10.1128/MRA.01602-18

**Published:** 2019-02-14

**Authors:** Marta Diez-Valcarce, Anna Montmayeur, Roman Tatusov, Jan Vinjé

**Affiliations:** a Oak Ridge Institute for Science and Education, Oak Ridge, Tennessee, USA; b Synergy, Atlanta, Georgia, USA; c IHRC, Atlanta, Georgia, USA; d Division of Viral Diseases, U.S. Centers for Disease Control and Prevention, Atlanta, Georgia, USA; KU Leuven

## Abstract

We report five near-complete sapovirus genome sequences, including GI.3, GII.2, and GII.6 and two novel GII.NA (not assigned) strains. These new sequences expand the collection of human sapoviruses, allowing for a more accurate phylogenetic analysis of circulating strains and for designing broadly reactive primers for their detection and typing.

## ANNOUNCEMENT

Human sapoviruses are genetically diverse viruses in the family *Caliciviridae* that can be classified into 19 genogroups, with viruses from genogroups GI, GII, GIV, and GV causing acute gastroenteritis (AGE) in humans. Viruses in these four genogroups can be further divided into 18 genotypes (1 – 3). We report the near-complete genomic sequences of three sapovirus genotypes (GI.3, GII.2, and GII.6) and two sequences representing a tentative new GII genotype, GII.NA1 (not assigned). These sapoviruses were detected in stool samples from hospitalized patients with AGE in 2006 (*n* = 2), an outpatient child with AGE in 2005 (*n* = 1), and children without diarrhea in 2008 (*n* = 2) in Southeast Kenya (Siaya County). The two GII.NA1 strains were obtained from a child with AGE symptoms in 2005 and from a child without diarrhea enrolled in a prospective case-control study in 2008 (4) and showed 94.9% nucleic acid identity with each other in the VP1 gene and 76% (GII.5) and 73% (GII.3) identity with the closest established sapovirus genotypes.

Viral nucleic acid was extracted from clarified 10% fecal suspensions, and viral metagenomics was performed as described previously (1). Briefly, virus particles were filtered and treated with nucleases, and nucleic acids were extracted using a QIAamp viral RNA minikit (Qiagen, Hilden, Germany). Complementary DNA (cDNA) synthesis by random amplification was performed using a sequence-independent single primer amplification (SISPA) protocol (5). PCR products were purified, and a 300-bp average fragment library was constructed using the Nextera XT DNA library preparation kit (Illumina, San Diego, CA). Samples were sequenced on an Illumina MiSeq system using a MiSeq reagent kit v2 (500 cycles, 2 × 250-bp paired-end). A total of 7,444,396 reads were generated, and quality trimming and filtering were performed using a custom bioinformatics pipeline (6, 7). Briefly, raw sequencing reads were ﬁltered to remove host DNA sequences using Bowtie 2 v2.1.0, and the primers and adapters were trimmed using Cutadapt v1.8. After filtering and trimming, 1,956,032 reads (20.6% of the total) remained, 403,533 of which (5.4%) were sapovirus. Complete genomes of human coxsackievirus (Siaya1927, Siaya2158, and Siaya2345), human enterovirus 99 (Siaya0494 and Siaya0506), and “Saffold” virus (Siaya0506) were also found.

Sapovirus genomes were assembled using the *de novo* assembler SPAdes (8) followed by reference mapping and gene annotation using Geneious v11.1.2 (Biomatters) (9). To compare the assembly results from different samples, we used the *de novo* assembly efficiency metric UG_50_% (10). All five samples have a value of ≥99.2% or 100.0%, demonstrating direct full-genome assembly.

Strain Hu/KE/2006/GI.3/Siaya1927 (missing 12 nucleotides [nt] of the 5′-untranscribed region [UTR] and 45 nt of open reading frame 1 [ORF1]) is 7,332 nt long. Strain Hu/KE/2006/GII.2/Siaya2158 (missing 13 nt of the 5′-UTR) is 7,382 nt long. Strain Hu/KE/2008/GII.NA1/Siaya0506 (complete genome) is 7,435 nt long. Strain Hu/KE/2005/GII.NA1/Siaya2345 (missing 8 nt of the 5′-UTR and 162 nt in ORF1) is 7,204 nt long. Strain Hu/KE/2008/GII.6/Siaya0494 (missing 13 nt of the 5′-UTR and 18 nt in ORF1) is 7,418 nt long.

### Data availability.

The genome sequences have been deposited in GenBank under the accession numbers MG012401 and MH922771 to MH922774, and the reads have been deposited in the Sequence Read Archive with the accession numbers SRR8446701 to SRR8446705.
